# Cardiac-specific Conditional Knockout of the 18-kDa Mitochondrial Translocator Protein Protects from Pressure Overload Induced Heart Failure

**DOI:** 10.1038/s41598-018-34451-2

**Published:** 2018-11-01

**Authors:** Phung N. Thai, Daniel J. Daugherty, Bert J. Frederich, Xiyuan Lu, Wenbin Deng, Donald M. Bers, Elena N. Dedkova, Saul Schaefer

**Affiliations:** 1 Department of Internal Medicine, Cardiovascular Medicine, University of California, Davis, CA USA; 2 Department of Biochemistry and Molecular Medicine, University of California, Davis, CA USA; 3 Department of Pharmacology, University of California, Davis, CA USA; 4 Department of Veterans Affairs, Northern California Health Care System, Mather, CA USA

## Abstract

Heart failure (HF) is characterized by abnormal mitochondrial calcium (Ca^2+^) handling, energy failure and impaired mitophagy resulting in contractile dysfunction and myocyte death. We have previously shown that the 18-kDa mitochondrial translocator protein of the outer mitochondrial membrane (TSPO) can modulate mitochondrial Ca^2+^ uptake. Experiments were designed to test the role of the TSPO in a murine pressure-overload model of HF induced by transverse aortic constriction (TAC). Conditional, cardiac-specific TSPO knockout (KO) mice were generated using the Cre-*lox*P system. TSPO-KO and wild-type (WT) mice underwent TAC for 8 weeks. TAC-induced HF significantly increased TSPO expression in WT mice, associated with a marked reduction in systolic function, mitochondrial Ca^2+^ uptake, complex I activity and energetics. In contrast, TSPO-KO mice undergoing TAC had preserved ejection fraction, and exhibited fewer clinical signs of HF and fibrosis. Mitochondrial Ca^2+^ uptake and energetics were restored in TSPO KO mice, associated with decreased ROS, improved complex I activity and preserved mitophagy. Thus, HF increases TSPO expression, while preventing this increase limits the progression of HF, preserves ATP production and decreases oxidative stress, thereby preventing metabolic failure. These findings suggest that pharmacological interventions directed at TSPO may provide novel therapeutics to prevent or treat HF.

## Introduction

Congestive heart failure (HF) is still one of the leading causes of death, despite advances in therapeutic options^1^. Current therapies aim at treating the systemic neurohormonal changes that occur during HF^2^, but none have been successfully developed to directly address cardiomyocyte function and viability. Understanding some of the key myocyte abnormalities in HF and developing specific therapies toward reversing these abnormalities can improve cardiac function and survival. Several abnormalities related to mitochondrial function have been observed in HF, including impairment of intracellular calcium (Ca^2+^) homeostasis^3,4^, reduced cellular respiration^5,6^, increase in the production of reactive oxygen species (ROS), and impaired mitochondrial quality control (mitophagy)^7^, all contributing to reduced contractile function^8^ and reduced myocyte survival^9^. These events all involve, in part, the proper communication between Ca^2+^ and mitochondria^10^.

During excitation-contraction coupling, the influx of Ca^2+^ through the voltage-gated Ca^2+^ channels triggers Ca^2+^-induced Ca^2+^ release from the ryanodine receptors (RyR2) on the sarcoplasmic reticulum (SR). Areas with high, localized concentrations of Ca^2+^, known as microdomains, form at the junction between the SR and mitochondria, and result in mitochondrial Ca^2+^ uptake^11,12^. In the normal heart, Ca^2+^ enters the mitochondria through the voltage-dependent anion channel (VDAC) on the outer mitochondrial membrane (OMM)^13^ before it passes through the mitochondrial Ca^2+^ uniporter (MCU) in the inner membrane^14^. It is known that mitochondrial Ca^2+^ serves as a regulator of energy production^6,14,15^. However, during HF, mitochondrial Ca^2+^ uptake is typically reduced^3,14,16^, which can impact the production of energy and contribute to the pathogenesis of HF.

The 18-kDa translocator protein (TSPO) is a five transmembrane domain protein that is primarily localized in the OMM and ubiquitously expressed throughout the body with elevated expression in steroid-synthesizing tissues, such as adrenal glands, gonads and the brain, as well as in the heart^17^. In addition to regulating steroidogenesis^18^, there is evidence that TSPO is involved in modulating oxidative stress^19^, and, critically, mitochondrial physiology and metabolism^20^. These latter effects include modulation of Ca^2+^ transport^21^, regulation of transcription of genes involved in mitochondrial electron transport chain, and thus ATP production^22^, and mitochondrial quality control^23^.

The above effects of TSPO could be explained via modulation of VDAC^23^, a ubiquitous channel located on the OMM which regulates the energy balance of mitochondria, serving as a common pathway for metabolite exchange between mitochondria and the cytoplasm^24^. Alterations in VDAC function, expressed as open-probability (P_o_), can impact oxidative phosphorylation by controlling the rate of adenine nucleotide exchange across the OMM^25^. Recent evidence has shown that expression of the TSPO is increased during stress conditions^26–30^, with the degree of expression reflecting the severity of the condition^31^. Thus, conditions which increase TSPO expression, such as HF, can modulate metabolism and affect cell survival through its effect on VDAC P_o_; however, no data exist on the role played by TSPO during HF development. Our previous work showed that altering TSPO activity with a pharmacologic ligand protoporphyrin IX (hemin) decreased Ca^2+^ uptake in mitochondria isolated from cardiac tissue by decreasing the open probability of VDAC^21^, which raises the possibility that TSPO may play a role in mitochondrial Ca^2+^ handling.

The objectives of the present study were to test the hypotheses that: a) HF increases expression of TSPO, with a concomitant decrease in inward mitochondrial Ca^2+^ entry and systolic function; and thus b) genetic modulation of the TSPO could normalize mitochondrial Ca^2+^ uptake and prevent the abnormalities in cardiac structure and function evident in HF, specifically by normalizing cellular energetics and redox balance inside cardiomyocytes. These hypotheses were tested using a cardiomyocyte-specific, conditional knockout of the TSPO in a murine model of pressure-overload HF. Our studies show for the first time that TSPO-KO animals were protected from the progression of HF when compared to wild-type (WT) controls. In concordance with this protection, TSPO-KO mice had elevated activity of the mitochondrial complex I which led to the reduction of the oxidative stress in TSPO-KO TAC mice, shifting redox balance toward a more reduced state and preserving the mitochondrial membrane potential (ΔΨ_m_). Accordingly, the TAC-induced decline in mitochondrial Ca^2+^ uptake was prevented in TSPO-KO vs. WT mice, with correspondent preservation of ATP generation. Furthermore, the signaling cascade activating the removal of damaged mitochondria via mitophagy was significantly impaired in WT TAC and restored in TSPO KO cardiomyocytes, thus contributing to improved mitochondrial function. Together, these data suggest that TSPO can be a potential pharmacological target to correct the abnormalities in mitochondrial Ca^2+^ transport and mitochondrial bioenergetics associated with HF.

## Results

### TSPO expression is upregulated in pressure overload-induced heart failure

**Figure 1:**
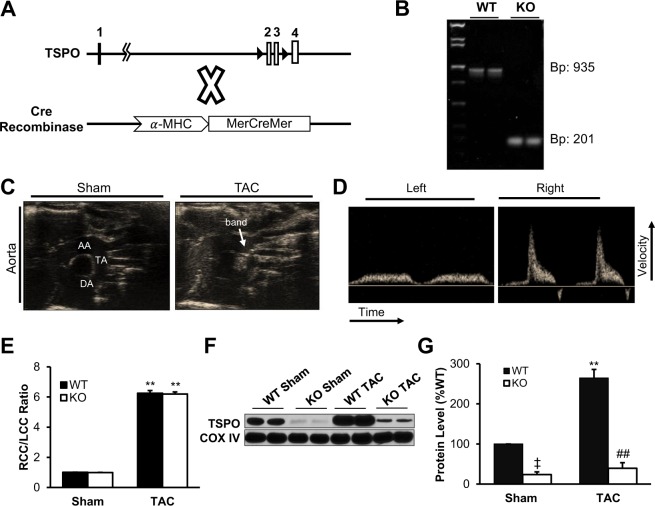
Transverse aortic constriction (TAC) significantly increased TSPO expression in WT mice, which was limited in the KO model. (**A**) A diagram of the Cre-LoxP system used to knockout the TSPO. Tamoxifen injection activated Cre recombinase, which excised exons 2 and 3 (arrowheads), thereby rendering the TSPO gene inactive. (**B**) A representative image of an agarose gel under UV lighting confirmed the *lox*P inserts flanking the TSPO, with WT displaying a band at 935 base pairs, and KO having a band at 201 base pairs. (**C**) Both WT mice and KO mice underwent transverse aortic constriction to induce heart failure. Representative images show a normal aorta, and an aorta that has been banded. AA = ascending aorta, TA = transverse aorta, DA = descending aorta. (**D**) After a week of TAC, Doppler flow velocity was taken in mice at the right common carotid (RCC) and left common carotid (LCC) to ensure the same degree of constriction. Representative images show tracings of flow velocity of the left and right common carotids in a WT TAC mouse. (**E**) The ratio of the RCC and LCC show similar degree of banding after TAC. (**F**) Representative western blot image for TSPO and COXIV (the loading control) in the 4 experimental groups [from the same gel] (**G**) revealed a significant increase in the TSPO expression level (normalized to COX IV) with TAC in the WT animals compared to sham controls and to KO TAC (n = 4 for all groups). Data expressed as mean ± SEM. ^‡^p < 0.01 vs WT sham, **p < 0.01 vs corresponding sham group, ^##^p < 0.01 vs WT TAC.

To examine the role of the TSPO, we generated cardiac-specific, conditional TSPO-KO mice using the Cre-*lox*P system as described in Methods and illustrated in Fig. 1A ^32^. DNA was taken from tails, amplified using polymerase chain reaction, processed through DNA electrophoresis, and visualized under UV lighting (Fig. 1B). WT animals showed bands at 935 base pairs (bp), while KO animals displayed bands at 201 bp confirming that the homozygous line for *Tspo* containing loxP sites was established.

To determine if the TSPO has a role in HF, we induced pressure-overload by surgical transverse aortic constriction (TAC) in 11-12 week-old mice. Representative images in Fig. 1C depict an aorta from a sham mouse (left) and an aorta from a mouse after one week of TAC (right). The degree of constriction was verified by color and pulse wave measurements of flow velocity in both carotid arteries one week after surgery (Fig. 1D,E), with values of 6.3 ± 0.4 mm/sec and 6.2 ± 0.4 mm/sec in wild-type TAC (WT TAC) and TSPO knockout TAC (KO TAC) mice, respectively (Fig. 1E). These measurements demonstrated identical increases in flow velocity following a week of TAC, indicating similar degrees of aortic constriction in the WT and KO animals. After 8 weeks, hearts were harvested and mitochondria were isolated from the whole hearts. As seen in Fig. 1F,G, 8 weeks of pressure-overload increased TSPO protein expression in the mitochondrial fraction of hearts from WT animals by 164% (p < 0.01, n = 4 hearts) over sham controls (WT sham, n = 4 hearts), an increase that was abrogated in the KO animals. As demonstrated by immunohistochemistry (Supplementary Fig. 1), very low TSPO levels are likely due mainly to TSPO in cells types other than cardiac myocytes where TSPO upregulation by inflammation has been reported previously^33,34^. These results provide the first evidence that cardiac TSPO is upregulated in failing hearts following TAC.

### TSPO ablation slows progression toward heart failure following TAC surgery

**Figure 2:**
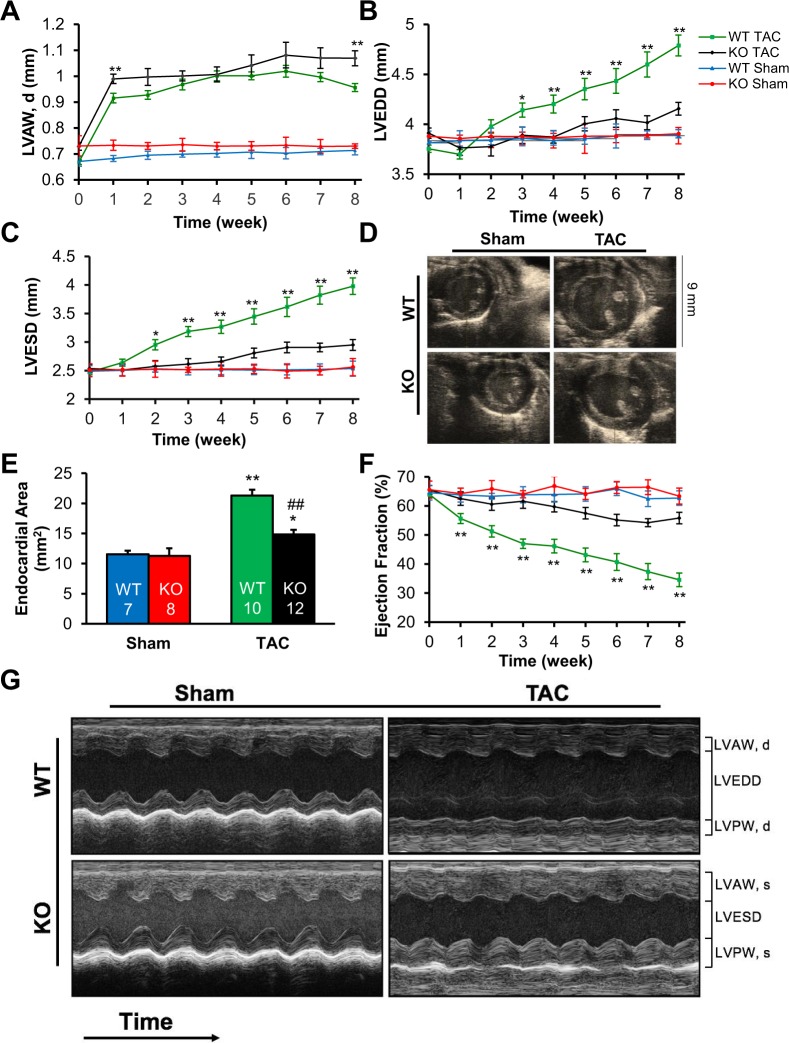
TSPO-KO TAC mice showed slower progression toward heart failure. (**A**) WT TAC mice and KO TAC mice had similar increases in wall thickness after 1 week, which was significantly different at 8 weeks. (**B**) Left ventricular end diastolic diameter (LVEDD) and (**C**) left ventricular end systolic diameter (LVESD) showed progressive cardiac dilation in WT TAC mice. (**D**) Representative images show m-mode short axis pictures of the heart in all four groups. (**E**) Endocardial area showed significantly higher cardiac dilation in WT TAC mice compared to KO TAC mice. (**F**) Systolic function, as measured by ejection fraction, showed that WT TAC mice had worse cardiac function starting at week 2. (**G**) Representative m-mode parasternal short axis images for all four groups after 8 weeks of sham or TAC surgery show that systolic dysfunction is most evident in WT TAC mice. n = 14, 9, 22, and 23 for WT sham, KO sham, WT TAC, and KO TAC respectively in (**A**–**C** and **F**). Data expressed as mean ± SEM. *p < 0.05 for WT TAC vs KO TAC, **p < 0.01 for WT TAC vs KO TAC.

To determine if TSPO has an adaptive or maladaptive effect on the development of pressure-overload HF, the anatomic and physiologic responses to pressure-overload were assessed weekly by echocardiography monitoring in unconscious mice. Sham animals, both WT and KO, did not exhibit morphologic or performance changes in the 8-week observation period after sham surgery. After TAC surgery, the increases in wall thickness after one week were similar in the WT and KO animals (Fig. 2A), which further supports a similar hemodynamic load in the two TAC groups. Following the initial hypertrophic compensation to the increase in afterload with TAC, the progression toward cardiac dilatation was significantly slower in KO TAC than WT TAC as manifest in both left ventricular end-diastolic dimension (LVEDD) and left ventricular end-systolic dimension (LVESD) (Fig. 2B,C). Similarly, WT TAC mice had more cardiac dilation than KO TAC mice, illustrated in the short axis m-mode images (Fig. 2D) and significantly higher ventricular cavity area (Fig. 2E, p < 0.01). These changes demonstrate the development of a dilated cardiomyopathy in the WT TAC animals, with relative preservation of chamber size in the KO TAC animals.

In parallel with the differences in wall thickness and ventricular dimensions, WT TAC animals developed reduced systolic function. As seen in Fig. 2F, WT animals subjected to TAC had a significant reduction in ejection fraction (EF) from 63.9 ± 1.0% at baseline to 34.5 ± 2.4% after 8 weeks (p < 0.01). In contrast, EF was minimally reduced in the KO TAC animals, from 65.5 ± 1.5% at baseline to 55.8 ± 2.0% after 8 weeks of TAC, resulting in a significant difference in final ejection fraction between the groups (p < 0.001, Fig. 2F, Table 1). Representative images of m-mode echocardiography demonstrate these changes in ventricular dimensions and ejection fraction (Fig. 2G). Together, these *in vivo* data indicate that knocking out TSPO limited HF development and preserved cardiac function with pressure overload.

**Table 1:** Echocardiographic parameters of WT and KO mice at 8 weeks after sham or TAC surgery.

	WT		KO		p-values		
	Sham (n = 14)	TAC (n = 22)	Sham (n = 9)	TAC (n = 23)	WT sham vs WT TAC	KO sham vs KO TAC	WT TAC vs KO TAC
**Echocardiography Measures**							
Heart Rate (bpm)	493 ± 41	523 ± 44	507 ± 41	517 ± 50	NS	NS	NS
Ejection Fraction (%)	62.7 ± 2.5	34.5 ± 2.4	63.4 ± 2.8	55.8 ± 2.0	<0.01	<0.05	<0.05
LVEDD (mm)	3.89 ± 0.08	4.79 ± 0.10	3.91 ± 0.04	4.16 ± 0.06	<0.01	<0.01	<0.01
LVESD (mm)	2.53 ± 0.13	3.98 ± 0.15	2.56 ± 0.15	2.95 ± 0.10	<0.01	<0.05	<0.05
LVAW,d (mm)	0.71 ± 0.02	0.96 ± 0.02	0.73 ± 0.01	1.07 ± 0.03	<0.01	<0.01	<0.01
LVAW,s (mm)	1.09 ± 0.03	1.21 ± 0.03	1.14 ± 0.02	1.47 ± 0.04	<0.01	<0.01	<0.01
LVPW,d (mm)	0.72 ± 0.02	0.95 ± 0.01	0.74 ± 0.02	1.06 ± 0.03	<0.01	<0.01	<0.01
LVPW,s (mm)	1.10 ± 0.03	1.21 ± 0.03	1.15 ± 0.03	1.47 ± 0.04	<0.01	<0.01	<0.01

Abbreviations: LVEDD, left ventricular end diastolic diameter; LVESD, left ventricular end systolic diameter; LVAWd, left ventricular anterior wall during diastole; LVAWs, left ventricular anterior wall during systole; LVPWd, left ventricular posterior wall during diastole; LVPWs, left ventricular posterior wall during systole. Data expressed as mean ± SEM.

### TSPO deletion protects the heart against pressure overload-induced hypertrophy and pathological cardiac remodeling

**Figure 3:**
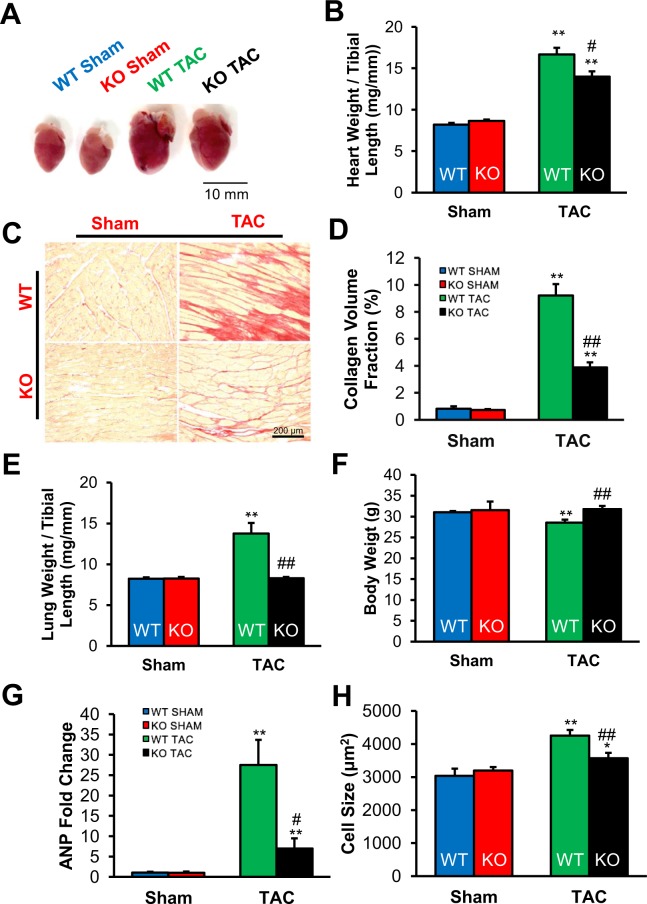
WT TAC mice developed worse dilated cardiomyopathy, and showed greater clinical manifestations of heart failure. (**A**) Representative heart pictures of all four groups show enlargement of hearts in TAC animals, with worse phenotype in the WT TAC heart including left atrial enlargement. (**B**) Postmortem analysis of heart weight-to-tibial length ratio indicated greater heart weight in WT TAC mice compared to hearts of either sham group or the KO TAC mice. (**C**) Representative photographs of transverse sections showed greater fibrosis in the hearts of WT TAC mice, which quantitatively had a (**D**) collagen volume fraction that was significantly higher than the fraction in KO TAC hearts; n = 4 hearts for all groups. (**E**) Postmortem lung weight-to-tibial length measurements showed evidence of pulmonary effusion in WT TAC mice. (**F**) WT TAC mice lost significantly more weight, while KO TAC mice gained weight, consistent with more severe HF in WT TAC. (**G**) Relative mRNA expression of ANP showed a much greater increase in atrial natriuretic peptide (ANP), a typical biomarker of heart failure, in WT TAC mice. (**H**) Cell size was significantly increased WT TAC cardiomycytes compared to WT and KO shams, and TSPO-KO TAC. n = 3–4 hearts. Data expressed as mean ± SEM. **p < 0.01 vs corresponding sham group, ^#^p < 0.05 vs WT TAC, ^##^p < 0.01 vs WT TAC.

To further characterize the severity of HF development, anatomical and histological analyses were conducted. Consistent with the echocardiographic findings, measurement of heart weight-to-tibial length (HW:TL) ratios revealed greater TAC-induced cardiac mass (due to greater dilation) in WT compared to KO animals (16.4 ± 1.0 vs 14.0 ± 1.2 mg/mm, p < 0.05, n = 14–18 hearts, Fig. 3A,B). Abnormalities in histology paralleled the gross anatomic changes, including higher collagen formation in WT TAC mice than KO TAC mice (9.2 ± 0.8% and 3.9 ± 0.4%, respectively; n = 3–4 hearts p < 0.01, Fig. 3C,D). These data show that KO TAC animals were relatively protected from TAC-induced cardiac remodeling.

In addition to the anatomical, functional, and structural changes, WT TAC mice had more clinical evidence of pulmonary congestion confirmed by a higher lung weight-to-tibial length (LW:TL) ratio (13.8 ± 1.9 mg/mm, n = 18) than their corresponding sham group (8.2 ± 0.2 mg/mm, n = 5, p < 0.01, Fig. 3E). In contrast, KO TAC mice did not experience a significant increase in the LW:TL ratio compared to sham controls. WT TAC mice exhibited significantly lower body weight (28.5 ± 0.7 g) compared to sham controls (31.0 ± 0.3 g, p < 0.01), while KO TAC mice did not (31.8 ± 0.8 g, Fig. 3F). Consistent with clinical signs of HF, there was a significant increase in atrial natriuretic peptide hormone (ANP) mRNA levels in both TAC groups when compared with their corresponding sham controls (Fig. 3G), although the WT TAC mice experienced a greater increase in ANP than did the KO TAC mice (p < 0.05). Furthermore, the size of cardiac myocytes, determined by confocal fluorescent microscopy imaging, was significantly increased in WT TAC (n = 80, P < 0.001) animals compared to WT shams (n = 56) (Fig. 3H). Cardiomyocyte cell size was also significantly smaller in KO TAC (p < 0.005, n = 66) compared to WT TAC, indicating that TSPO-KO also limited pathological myocyte hypertrophy. Taken together, TSPO KO mice develop compensated hypertrophy in response to TAC, whereas WT mice progress fully to HF in the same 8 week period (based on EF, chamber size, cardiac fibrosis, lung weight and ANP expression).

### Cardiac TSPO-KO prevented TAC-induced impairment of mitochondrial Ca^2+^ uptake

**Figure 4:**
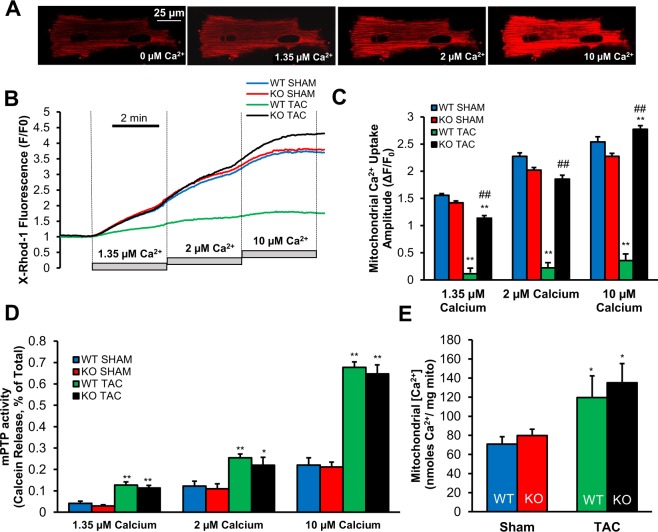
Cardiac specific TSPO silencing restored impaired mitochondrial Ca^2+^ uptake in HF. (**A**) Representative confocal images of cardiomyocytes loaded with X-Rhod-1 after permeabilization and subsequent increase in extramitochondrial Ca^2+^. (**B**) Representative tracings of mitochondrial Ca^2+^ uptake in permeabilized cardiomyocytes using the calcium indicator X-Rhod-1 after the addition of 1.35 µM Ca^2+^, 2 µM Ca^2+^, and 10 µM Ca^2+^. (**C**) Mean group data showing mitochondrial Ca^2+^ uptake amplitude in the four groups. Data expressed as mean ± SEM. n = 3 hearts for each group, 10–17 cells. *p < 0.05 vs corresponding sham group, **p < 0.01 vs corresponding sham group, ^##^p < 0.01 vs WT TAC. (**D**) Measurement of mPTP activity in permeabilized cardiomyocytes by calcein release from mitochondria upon elevation of [Ca^2+^]_em_ from 0 to 1.35 µM Ca^2+^, 2 µM Ca^2+^, and 10 µM Ca^2^ in four experimental groups. Summary data indicates an increase in mPTP activity in all three Ca^2+^ conditions in both TAC groups when compared with their sham counterparts. However, there was no significant difference in mPTP activity between the two TAC groups at all Ca^2+^ levels. n = 3 hearts, 10–25 cells. Data expressed as mean ± SEM. *p < 0.05 vs corresponding sham group, **p < 0.01 vs corresponding sham group, ^†^p < 0.05 vs WT sham. (**E**) Measurement of basal free Ca^2+^ in isolated mitochondria from 4 experimental groups revealed that there was a small increase (P < 0.05) in TAC groups compared to the corresponding sham controls (n = 6 in each group), however no difference was observed between WT (n = 10) and KO TAC (n = 11, P = 0.3).

HF has been characterized by an alteration in mitochondrial Ca^2+^ uptake^14,16^. Our previous work using hemin-induced TSPO activation suggested that TSPO can suppress mitochondrial Ca^2+^ uptake^21^. The current study used the WT vs. TSPO-KO to test whether the dramatic rise in TSPO in TAC might contribute to reduced mitochondrial Ca^2+^ in TAC. Mitochondrial Ca^2+^ uptake amplitude (assessed using X-Rhod-1), was similar in myocytes from WT and TSPO-KO mice under sham conditions (Fig. 4A–C). However, TAC caused a dramatic 86–93% reduction in WT mouse mitochondrial Ca^2+^ uptake at the three [Ca^2+^] studied (Fig. 4B,C). In sharp contrast, TSPO-KO mice exhibited nearly unaltered mitochondrial Ca^2+^ uptake after TAC (<20% reduction at 1.35 µM Ca^2+^, no change at 2 µM Ca^2+^ and slight increase at 10 µM Ca^2+^). Moreover, after TAC, mitochondrial Ca^2+^ uptake in KO myocytes was 7.7–9.5-fold higher than that in WT (black vs. green bars in Fig. 4C). We conclude that normal physiological levels of TSPO expression do not limit acute mitochondrial Ca^2+^ uptake, but that the high levels of TSPO in TAC-WT may contribute to reduced mitochondrial Ca^2+^ uptake. Thus, preventing the TAC-induced increase in TSPO expression in HF by conditional cardio-specific silencing of TSPO preserved mitochondrial Ca^2+^ uptake.

Since opening of the mitochondrial permeability transition pore (mPTP) has been associated with Ca^2+^ overload^35^, increased ROS^36^ and resultant myocyte death, the effect of TSPO-KO on mPTP was examined using calcein release from mitochondria^37–39^. Figure 4D shows that both WT and TSPO-KO sham had a similar low mPTP activity that increased progressively (and similarly) as [Ca^2+^] was raised. Myocytes from TAC hearts (both WT and TSPO-KO) exhibited higher mPTP opening at any given [Ca^2+^], but no differences in mPTP activity were seen between TSPO-KO and WT TAC myocytes (Fig. 4D). These data indicate that TAC caused increased mPTP activation, but TSPO ablation did not prevent this effect. Of note, the high mPTP rate in WT TAC mitochondria occurred at a lower free mitochondrial [Ca^2+^] (Fig. 4C), so this might reflect a higher ROS level in the HF myocytes that would promote mPTP at lower free [Ca^2+^]. We also measured basal levels of total mitochondrial Ca^2+^ content measured in isolated mitochondria lysed in 0Ca^2+^/0Na^+^ conditions. Baseline total mitochondrial Ca^2+^ was elevated in both WT TAC and KO TAC compared to their corresponding sham controls (Fig. 4E); as higher total mitochondrial Ca^2+^ content also promotes mPTP^40^, this might explain the similar mPTP in WT TAC vs. KO TAC mitochondria.

### ATP generation was reduced in WT TAC mice, but preserved in TSPO-KO TAC mice

**Figure 5:**
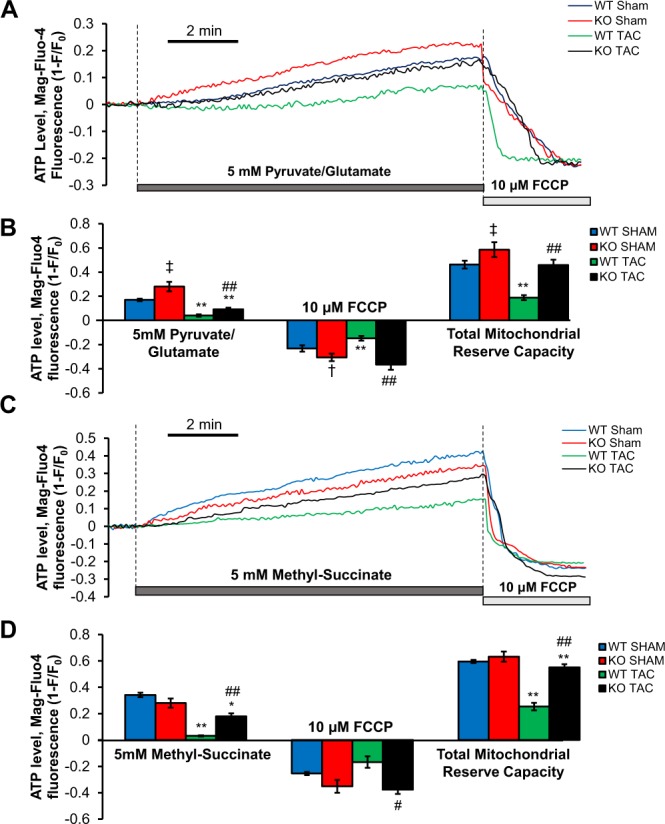
ATP generation in WT TAC mice was reduced, but was preserved in KO TAC mice. (**A**) Representative tracings of normalized inverted fluorescent intensities of all 4 groups in the presence of 5 mM pyruvate/glutamate. 10 μM FCCP was added to uncouple mitochondria. (**B**) ATP levels and total mitochondrial reserve capacity were greater in the KO TAC cardiomyocytes than the WT TAC cardiomyocytes in complex I mediated respiration. n = 3 hearts, 9–14 cells. (**C**) Representative tracings of normalized inverted fluorescent intensities of all 4 groups in the presence of 5 mM of methyl-succinate. (**D**) ATP levels in KO TAC cardiomyocytes were preserved in complex II mediated respiration. n = 3 hearts, 6–8 cells. Data expressed as mean ± SEM. *p < 0.05 vs corresponding sham group, **p < 0.01 vs corresponding sham group, ^†^p < 0.05 vs WT sham, ^‡^p < 0.01 vs WT sham, ^#^p < 0.05 vs WT TAC, ^##^p < 0.01 vs WT TAC.

Ca^2+^ entry into the mitochondria can regulate energy production via activation of several key dehydrogenases^3,14,16^, and enhancing mitochondrial Ca^2+^ in the context of HF can maintain NADH oxidation^3^ and, hence ATP production^6^. To assess substrate-dependent [ATP] changes in intact cells under substrate conditions which activate complex I (pyruvate/glutamate - Fig. 5A,B) or complex II (succinate - Fig. 5C,D), we monitored changes in [ATP]_i_ indirectly using [Mg^2+^]-sensitive dye mag-fluo-4 (free [Mg^2+^]_i_ rises as [Mg-ATP] declines)^37^. Substrate addition led to an increase in ATP level above baseline in all groups, which was significantly higher in KO sham cardiomyocytes compared to WT sham cardiomyocytes in the presence of pyruvate/glutamate (p < 0.01), but not in the presence of methyl-succinate. This finding suggests that complex I mediated respiration drove significantly more ATP production in KO sham cardiomyocytes vs. WT. With methyl-succinate to drive complex II mediated respiration, there was no difference between WT and KO shams (Fig. 5C,D). In WT mouse myocytes, TAC nearly abolished the ATP rise induced by either pyruvate and glutamate or methyl-succinate. However, in TSPO-KO TAC myocytes, ATP levels achieved were higher than in the WT animals after TAC for either substrate (albeit still lower than sham).

Corresponding to the improved ATP levels in the TSPO-KO TAC animals (vs. WT TAC), TSPO ablation resulted in restoration of the mitochondrial reserve capacity in TSPO-KO TAC cardiomyocytes utilizing substrate for either mitochondrial complex I or II (Fig. 5B,D, right panels). Furthermore, the reserve capacity was higher in sham TSPO-KO vs. WT mice, but only for pyruvate/glutamate as substrates for mitochondrial complex I.

Together, these data confirm the adverse effect of TAC-induced heart failure on ATP production^37,41^ and suggest that the effects of the TSPO-KO cardioprotection after TAC may be mediated by enhancement of energy production via complex I.

### Mitochondrial FAD redox potential remained more reduced in TSPO-KO TAC animals

**Figure 6:**
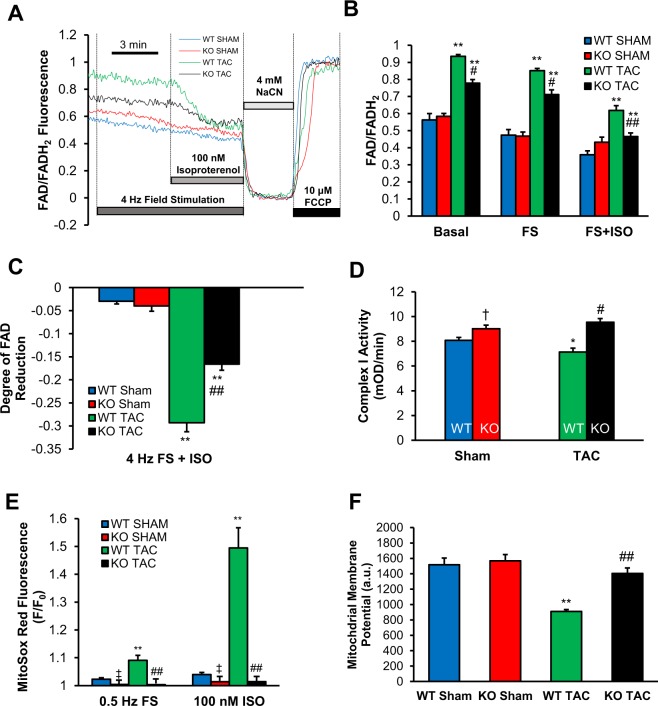
Reactive oxygen species production was elevated in WT TAC cardiomyocytes, and decreased significantly in KO cardiomyocytes, while mitochondrial redox potential remained more reduced in KO cardiomyocytes. (**A**) Representative traces show the FAD/FADH_2_ fluorescence intensity in intact ventricular myocytes following electrical field stimulation at 4 Hz and subsequent application of 100 nM isoproterenol, 4 mM NaCN, and 10 μM FCCP. Application of 4 mM NaCN induces maximal reduction of FAD, and application of 10 µM FCCP induces maximal oxidation of FADH_2_. (**B**) The fluorescence intensity ratio represents the shift of redox potential from the reduced state (lower ratio) to more oxidized state (higher ratio) following TAC or the tendency to acquire electrons. (**C**) Quantification of the FAD/FADH_2_ ration revealed inability of WT mice to maintain the redox potential following isoproterenol stimulation. (**D**) Complex I activity was severely impaired in WT TAC compared to sham groups but restored in TSPO-KO TAC. (**E**) Shown are amplitudes of the MitoSOX Red fluorescence (reflects mitochondrial superoxide generation) measured at the end of electrical field stimulation at 0.5 Hz and subsequent stimulation with 100 nM isoproterenol. There was an increase in ROS generation in WT TAC cardiomyocytes, and decrease ROS generation in both KO groups. (**F**) Shown are measurements of the basal mitochondrial membrane potential in 4 experimental groups. As shown WT TAC mice have mitochondria with severely depolarized ΔΨ_m_. n = 3 animals, 17–39 cells. Data expressed as mean ± SEM. *p < 0.05 vs corresponding sham group, **p < 0.01 vs corresponding sham group, ^†^p < 0.05 vs WT sham, ^‡^p < 0.01 vs WT sham, ^#^p < 0.05 vs WT TAC, ^##^p < 0.01 vs WT TAC.

It has been previously reported that restoring mitochondrial Ca^2+^ uptake in the failing hearts plays an essential role in maintaining the NADH/FAD redox balance^3^. We therefore hypothesized that the inability of cells to maintain normal mitochondrial Ca^2+^ uptake in WT TAC mice could contribute to dysregulation of the redox potential and electron flux. To test that hypothesis, we monitored mitochondrial redox potential by FAD/FADH_2_ autofluorescence under conditions of increased cell work induced by electrical field stimulation at 4 Hz and subsequent isoproterenol (ISO) stimulation (Fig. 6A).

Quantitative analysis in WT TAC cardiomyocytes revealed nearly maximal FAD/FADH_2_ ratio (oxidized) at basal conditions, significantly more oxidized than WT sham (0.94 ± 0.01, n = 10 vs 0.56 ± 0.04, n = 14, p < 0.001, Fig. 6B). Most importantly, we found WT TAC myocytes were not able to maintain a redox state when cells were exposed to high work load induced by 4 Hz stimulation (FS) with isoproterenol. That is, the degree of FAD reduction, which inversely correlates with NADH oxidation, was nearly ten-fold higher in WT TAC cardiomyocytes vs. WT sham cardiomyocytes (Fig. 6C). In TSPO-KO TAC cardiomyocytes, there was still significant reduction in FAD/FADH_2_ with stimulation plus isoproterenol, but this was significantly smaller when compared to WT TAC myocytes (−0.16 ± 0.1, n = 10 vs −0.29 ± 0.2, n = 10, p < 0.001). In both WT and KO sham cardiomyocytes, FAD/FADH_2_ levels were well maintained during 4 Hz FS and ISO stimulation (Fig. 6C). These data suggest a possible impairment in the electron flow at the level of the respiratory chain in WT TAC mice which was partially restored in KO TAC mice.

### Complex I activity was impaired in WT TAC and associated with decreased mitochondrial membrane potential and enhanced ROS generation

We^41^ and others^42^ previously reported that electron transfer during HF is impaired at the level of the mitochondrial complex I, which can increase ROS accumulation. We found that WT TAC mice had reduced complex I activity compared to WT sham mice (7.4 ± 0.3 vs 8.5 ± 0.2 mOD/min, p < 0.01), whereas KO TAC mice had preservation of complex I activity (similar to KO sham). Importantly, we found that complex I activity after TAC was much higher in TSPO-KO vs. WT mice (9.5 ± 0.3 vs 7.4 ± 0.3 mOD/min, p < 0.01, Fig. 6D). Together, these data show that complex I activity is impaired in WT TAC mice, but preserved in KO TAC mice.

To examine whether KO TAC mice have less ROS generation compared to WT TAC mice, superoxide generation was measured in cardiomyocytes using MitoSOX Red fluorescent dye^38,39^. Figure 6E shows that WT cardiomyocytes had significantly higher superoxide production both under sham conditions and after TAC, following electrical field stimulation at 0.5 Hz or beta-adrenergic stimulation with 100 nM isoproterenol. Notably, superoxide generation in cardiomyocytes from KO TAC animals was similar to that of sham animals (1.02 ± 0.01, n = 10 vs 1.04 ± 0.01, n = 19, p = 0.1). Together, these data show that ROS generation was increased by HF in WT TAC mice, and limited to control levels in TSPO-KO animals.

Furthermore, we found that mitochondrial membrane potential (ΔΨ_m_) was significantly decreased in WT TAC cardiomyocytes, but not in TSPO-KO TAC (Fig. 6F). Application of 10 µM rotenone, a mitochondrial complex I inhibitor, led to a significant decrease in ΔΨ_m_ in WT sham from 1516 ± 89 to 858 ± 43 a.u., n = 39, p < 0.001 but not in WT TAC myocytes where basal level was already at 911 ± 24 a.u., n = 17 (not shown), confirming that the inability of mitochondria to maintain ΔΨ_m_ in HF was related to impairment of the mitochondrial complex I. In TSPO-KO TAC mice, basal levels of ΔΨ_m_ were significantly closer (1404 ± 72 a.u., n = 24) to that observed in sham controls (1567 ± 81 a.u., n = 39). We conclude that TSPO ablation improved mitochondrial oxidative phosphorylation via restoration of the electron flux flow at the level of the mitochondrial complex I.

### Mitophagy was impaired in WT TAC, but preserved in KO TAC cardiomyocytes

Efficient degradation and replacement of dysfunctional mitochondria is essential for cardiac myocyte survival. Damaged mitochondria are eliminated via mitochondrial autophagy or mitophagy. Upon mitochondrial depolarization, the serine/threonine kinase PTEN-inducible kinase 1 (PINK1) accumulates on the mitochondrial surface, recruiting the E3 ubiquitin ligase (Parkin) to the outer mitochondrial membrane from the cytosol. Parkin then polyubiquitinates mitochondrial membrane proteins, marking them for degradation and recognition by the adaptor protein SQSTM1/p62. SQSTM1/p62 binds to the microtubule-associated protein 1A/1B-light chain 3 (LC3) on the autophagosome and to the ubiquitinated mitochondrial proteins, tethering the autophagosome to the mitochondrion^7^. The autophagosome then engulfs the mitochondrion and fuses with lysosomes, allowing mitochondrial proteins to be degraded and recycled.

**Figure 7:**
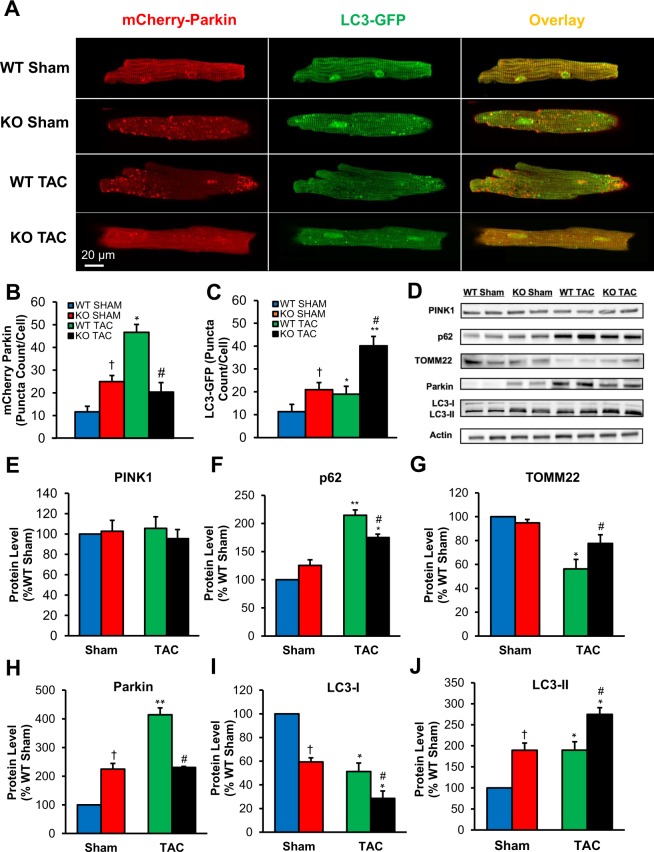
Impaired mitophagy in WT TAC cardiomyocytes, and preserved mitophagy in KO TAC cardiomoycoytes. (**A**) Representative cardiomyocyte images from all four groups showing cells with mCherry-Parkin, LC3-GFP, and the combined images of both. (**B**) Quantitative analysis (puncta count/cell) revealed the increase in m-Cherry Parkin in WT TAC cardiomyocytes, but not in KO TAC cardiomyocytes. (**C**) Further quantitative analysis for LC3-GFP (autophagosome formation indication) showed that there was actually a decrease in LC3-GFP in WT TAC cardiomyocytes, while LC3-GFP in KO TAC cardiomyocytes increased, suggesting that there was increase in mitochondrial autophagy in KO TAC cardiomyocytes and not in WT TAC cardiomyocytes. (**D**) Shown are representative Western blots of the proteins participating in signaling cascade that activates mitophagy in cardiac myocytes: PINK-1, SQSTM1/p62, TOMM22, and LC3-I/LC3-II in cell lysates from four experimental groups. Actin staining was used to verify equal protein loading for each experimental group. (**E**) Quantification of PINK-1 protein expression normalized to WT sham levels which are taken as 100%. (**F**) Quantification of SQSTM1/p62 protein expression normalized to WT sham levels which are taken as 100%. (**G**) Quantification of TOMM22 protein expression normalized to WT sham levels which are taken as 100%. (**H**) Quantification of SQSTM1/p62 protein expression normalized to WT sham levels which are taken as 100%. (**I**) Quantification of LC3-I protein expression normalized to WT sham levels which are taken as 100%. (**J**) Quantification of LC3-I conversion to LC3-II protein expression normalized to WT sham levels which are taken as 100%. n = 3–5 animals, 8–15 cells used for each group in confocal microscopy experiments. Data expressed as mean ± SEM. *p < 0.05 vs corresponding sham group, **p < 0.01 vs corresponding sham group, ^†^p < 0.05 vs WT sham, ^‡^p < 0.01 vs WT sham, ^#^p < 0.05 vs WT TAC, ^##^p < 0.01 vs WT TAC.

We evaluated mitophagy by monitoring mitochondrial Parkin accumulation and LC3-mediated autophagosome formation in cardiac myocytes using confocal microscopy. As shown in Fig. 7A–C, WT TAC myocytes exhibited increased mCherry-Parkin puncta accumulation (marking damaged mitochondria) which was not accompanied by an increase in LC3-mediated autophagosome formation. This indicates impairment of mitophagy flux in TAC-induced HF. In contrast, mitophagy flux was enhanced in TSPO KO TAC mice (i.e. lower accumulation of Parkin puncta, but more LC3-autophagosomes). While lower levels of both mCherry-Parkin and LC3-GFP puncta were observed in WT sham myocytes, both were increased in TSPO KO sham myocytes, consistent with potential TSPO-induced suppression of mitophagy even in control myocytes. These results were further confirmed by blots assessing key proteins involved in mitophagic signaling. There were no significant changes in PINK-1 expression among the groups (Fig. 7D,E); however, WT TAC had reduced expression of TOMM22, which imports PINK1 into the mitochondrial matrix for cleavage by mitochondrial processing proteinase (MPP) and presenilin-associated rhomboid-like (PARL; Fig. 7D,G). Conversely, expression of the adaptor protein SQSTM1/p62 was greatest in WT TAC, but this did not correlate with LC3-II levels (Fig. 7D,G). In TSPO KO TAC myocytes, TOMM22 levels were partially increased while p62 levels were lower compared to WT TAC samples; however, the conversion of LC3-I to LC3-II was improved (Fig. 7D–G). As LC3-II is a lipidated form of LC3-I that localizes on phagophores and autophagosomes, the LC3-II/LC3-I ratio is indicative of mitophagic flux and was highest in KO TAC myocytes. Taken together, these measurements demonstrate that there was an increase in the removal of damaged mitochondria via mitophagy in KO TAC cardiomyocytes, but that this mitophagic flux was impaired in WT TAC cardiomyocytes.

## Discussion

Our results yield several key findings with respect TSPO function in pressure-overload induced heart failure (TAC). First, we demonstrated that TAC resulted in a 3-fold increase in expression of the TSPO in WT animals (Fig. 1F,G), an upregulation of TSPO that parallels that seen in cardiac ischemia and reperfusion^29^ and under stress conditions in other tissues^27,28,30^. Pressure overload for 8 weeks in WT animals resulted in the expected changes in cardiac morphology, function, and metabolism associated with HF (see Figs 2 and 3, and Table 1). These included an increase in cardiac chamber size, a 46% decrease in ejection fraction, an increase in myocyte hypertrophy and fibrosis, and clinical signs of HF such as pulmonary congestion and an increase in atrial natriuretic peptide.

Second, this study demonstrated that cardiac-specific conditional KO of TSPO substantially abrogated the deleterious effects of TAC. By preventing TSPO upregulation during TAC, there was well-preserved left ventricular function (only 15 vs. 46% reduction in ejection fraction in TSPO-KO vs. WT animals). In addition, TSPO-KO decreased associated HF markers, with lower levels of ventricular dilation, pulmonary congestion, ANP levels and extents of cardiac fibrosis and hypertrophy.

Third, TSPO-KO had several salutary effects on mitochondrial function in TAC mice, including normalization of mitochondrial Ca^2+^ uptake (Fig. 4) and ATP production (Fig. 5), lower production of ROS, maintenance of mitochondrial membrane potential, redox state (Fig. 6) and preservation of mitophagy (Fig. 7). Remarkably, the TSPO-KO at baseline (sham) was not appreciably different than the WT sham in any of the parameters that we assessed. We conclude from this observation that physiological levels of TSPO expression do not have overt negative effects, but that the dramatic upregulation of TSPO in TAC (and other stress conditions) has impactful negative effects that worsen HF progression and function. It is possible that the TSPO upregulation exceeds the stoichiometry of its normal physiological targets or partners, allowing it to interact preferentially with new targets that mediate pathological effects.

As a modulator of VDAC^43^, TSPO can affect inward mitochondrial Ca^2+^ flux. We have previously shown that the TSPO ligand protoporphyrin IX (hemin) reduced Ca^2+^ uptake in isolated mitochondria^21^, as was shown with another TSPO ligand PK11195^44,45^. Upregulation of TSPO expression during HF coincided with a decrease in mitochondrial Ca^2+^ uptake, which was normalized in TSPO-KO mice (Fig. 4). Together, these data are consistent with excess TSPO being a negative modulator of mitochondrial Ca^2+^ entry, potentially by interacting with and modulating VDAC open probability^21^. VDAC operates at the OMM, and controls Ca^2+^ entry into the intermembrane space in series with the mitochondrial Ca^2+^ uniporter (MCU) on the IMM^46,47^. A recent study^13^ demonstrated that reducing Ca^2+^ flux via VDAC severely affected heart contractility in zebra fish, while interventions to increase Ca^2+^ flux through the OMM restored normal contraction of the heart. These data highlight the possible impact of OMM proteins such as VDAC and TSPO in the regulation of mitochondrial Ca^2+^ uptake and cardiac function.

The remarkable beneficial effects of the TSPO-KO (at the mitochondrial, cardiac structure and functional level) are consistent with TSPO upregulation being a central amplifier of the progression from compensated hypertrophy (seen in TSPO-KO mice after TAC) to more full-blown HF (seen in WT TAC). A primary effect of TSPO overexpression to inhibit mitochondrial Ca^2+^ uptake could lead to most of the mitochondrial sequelae (on ATP and ROS production redox state and membrane potential), but we cannot rule out additional primary TSPO effects. Better maintenance of mitochondrial and energetic function by TSPO-KO, as seen here during TAC, could maintain cardiac function and slow the progression to HF^48^. Notably, the TSPO-KO TAC mice (vs. WT TAC) had higher respiratory reserve values and higher activity of the mitochondrial complex I, indicating preservation of mitochondrial bioenergetics. These findings agree with those of Gatliff *et al*.^49^ who showed that CF35 cells with siRNA-induced TSPO knockdown showed greater mitochondrial ATP production compared to control cells, while an overexpression of TSPO decreased ATP. Taken together, our data show that TSPO-KO mice were resistant to the progression of HF, and this protective effect was most likely due to a combination of enhanced mitochondrial Ca^2+^ uptake and energy production.

This situation contrasts with the known deleterious effect of mitochondrial Ca^2+^ overload under acute ischemia/reperfusion^39,50^. With ischemia/reperfusion, significant Ca^2+^ overload during reperfusion, mediated by sarcolemmal Na^+^/Ca^2+^ exchanger^50,51^, can result in opening of the mitochondrial permeability transition (mPTP), leading to mitochondrial swelling, membrane rupture and myocyte death. In contrast to the detrimental effect of Ca^2+^ overload under this acute pathologic challenge, the better maintained physiological mitochondrial Ca^2+^ uptake during chronic TAC in TSPO-KO promoted improved mitochondrial energetics and cardiac function. Notably, mPTP, which is a key turning point in above ischemia/reperfusion damage, was not influenced by TSPO-KO (Fig. 4D). Consistent with these findings, cyclosporine A treatment, a known mPTP inhibitor, while limiting deterioration of mitochondrial function in HF animals, was not associated with a commensurate improvement in cardiomyocyte Ca^2+^ handling or contractility^52^.

HF has been shown to result in increased oxidative stress^38,53^ and a higher FAD/FADH_2_ ratio^54^. These abnormalities have been shown to result in greater myocyte loss and dysfunction^55^, observations consistent with those observed with progressive HF in the WT TAC animals in the current study. In contrast, TSPO-KO mice demonstrated preserved function and less fibrosis, coupled with lower oxidative stress. The lower oxidative stress in the KO animals is likely due to the preservation of complex I activity^38,42^, as well as the higher ATP generation resulting in activity of key Ca^2+^-dependent dehydrogenases^56^.

It is increasingly clear that cardiac mitochondrial ROS production is dynamically regulated by Ca^2+^, ADP and the redox state of mitochondrial pyridine nucleotides^57^. One aspect is that ROS production can be increased by mitochondrial [Ca^2+^] that is either too high or too low (involving NADH, NADPH and FADH_2_). This “Redox-optimized ROS balance” hypothesis^58,59^ postulates that the physiological steady state in cardiac mitochondria is tuned to an intermediate redox state that prevents excessive ROS formation at the ETC under highly reduced conditions^60–62^, but also maintains anti-oxidative capacity under highly oxidized conditions^58,59,63^. Our data fit with this concept, as basal FAD/FADH_2_ ratio was 0.56 under sham conditions, with minimal ROS generation. During HF, however, Ca^2+^ uptake was depressed and FAD/FADH_2_ was severely oxidized, with a concordant increase in ROS generation. TSPO ablation restored normal Ca^2+^ flux to mitochondria, improved complex I activity, shifting redox index toward an “intermediate state” and reduction in ROS generation (see Fig. 6), which ultimately resulted in the improvement of cardiac function *in vivo*.

Our results are also consistent with results of Gatliff *et al*.^23^, in mouse embryonic fibroblasts and canine mammary gland epithelia. They concluded that TSPO, via its interaction with VDAC1, causes mitochondrial uncoupling, increased ROS production and inhibition of mitophagy by limiting the Parkin-dependent ubiquination. This fits with our data from KO TAC mitochondria, myocytes and hearts, where mitochondrial coupling in complex I was enhanced, ROS levels were lower and mitophagy was preserved vs. WT TAC. Moreover, this enhanced mitochondrial health and Ca^2+^ handling in TSPO KO hearts was associated with dramatically improved cardiac functional response to pressure overload, preventing transition from compensated hypertrophy to HF. These results should also further stimulate studies aimed at clarifying the detailed molecular mechanisms by which elevated levels of the OMM protein TSPO in HF mediate multiple pathological changes in mitochondrial function and turnover.

## Limitations

As with any murine animal model, one can neither extrapolate these findings to other HF causes (e.g. myocardial infarction, anthracycline toxicity), nor to human or large animal models. TSPO has numerous reported effects on steroid transport and lipid metabolism^64^, porphyrin metabolism^65^, and fatty acid oxidation^66^. While beyond our present scope, one or more of these pathways could also be involved in the protective effect of TSPO-KO in HF besides the mechanisms reported in this study.

## Conclusion

Taken together, these experiments have shown that HF induced by pressure overload resulted in increased expression of TSPO. Abrogating this increase by genetic ablation resulted in cardioprotection reflected by preserved ventricular function, cellular histology and clinical signs of heart failure. This cardioprotective effect was associated with preserved mitochondrial Ca^2+^ uptake and complex I-mediated respiration leading to the normalization of ATP production oxidative stress and mitochondrial quality control. These findings are the first observations that modulation of the mitochondrial outer membrane translocator protein could be a target for the prevention or treatment of HF.

## Methods

All protocols were in accordance with the Guide for the Care and Use of Laboratory Animals published by the National Institutes of Health (NIH Publication NO. 85-23, revised 1996) and approved by the University of California Davis Institutional Animal Care and Use Committee.

### Model of pressure overload hypertrophy/heart failure

C57Bl/6J mice were obtained from The Jackson Laboratory (Sacramento, CA). The aorta was surgically constricted as previously described^67^. A 5-mm thoracotomy was made and the transverse aorta was isolated and a suture looped around a blunt 27-gauge needle, yielding a transverse aortic diameter of approximately 0.4-mm. The sham procedure was identical except that the aorta was not ligated.

### Generation of cardiac-specific conditional TSPO^−/−^ mice

TSPO-floxed mice were produced in conjunction with the Mouse Biology program at UC Davis. TSPO-floxed mice were bred with mice containing the Tg(Myh6-cre)1Jmk/J gene (The Jackson Laboratory, Sacramento CA), in order to generate inducible, cardiac-specific, conditional KO mice. To genetically determine if TSPO was floxed, DNA was taken from tails, amplified using polymerase chain reaction, processed through DNA electrophoresis, and visualized under UV lighting. The *Tspo* gene had normal expression until Cre-mediated deletion of exons 2 and 3, induced by a single intraperitoneal injection of 40 mg/kg of tamoxifen (dissolved in sunflower oil and 100% ethanol at a 10:1 ratio). This recombination created a frame-shift mutation and a premature stop, which rendered the *Tspo* gene inactive. Exons 2 and 3 in the TSPO gene were flanked with *lox*P sites. This vector construct was inserted into C57BL/6 embryonic stem cells, which were subsequently screened through polymerase chain reaction (PCR) and DNA electrophoresis. Mice that had *lox*P sites were bred with each other to form TSPO^flox/flox^ mice. Homologous TSPO^flox/flox^ mice were then bred with mice containing the Tg(Myh6-cre)1Jmk/J gene. The Tg(Myh6-cre)1 Jmk/J gene controls the expression of Cre recombinase by the mouse myosin, heavy polypeptide 6 (Myh6). Animals that were homologous for TSPO^flof/flox^ and had the inducible Cre recombinase were considered to be KOs.

### Quantitative Real-Time PCR (RT-PCR)

Total RNA was extracted using a RNA fibrous tissue mini kit (Qiagen, Hilden Germany) and cDNA synthesized from 1 µg of RNA using the iScript^TM^ cDNA synthesis kit (Bio-Rad, Hercules, CA). qRT-PCR was performed with commercial and customized TaqMan probes (Life Technologies^TM^, ThermoFisher Scientific, Grand Island NY. Target gene mRNA level of ANP was normalized to GAPDH which served as housekeeping gene for comparison.

### Echocardiography

Ventricular function was monitored using 2D echocardiography on a weekly basis using a Visualsonics Vevo 2100 echocardiography machine and a MS 550D probe (22–55 MHz). Animals were anesthetized using isoflurane (0.75–1%). 2D echocardiography was performed at weekly intervals until the mice were ready to be sacrificed after 8 weeks of sham or TAC operation. Ejection fraction was determined from 2D images by manual determination of endocardium and epicardium from end-diastolic and end-systolic images.

### Morphometric Measurements

Eight weeks after surgery, mice were anaesthetized with isoflurane and sacrificed by cervical dislocation. Hearts were excised, weighed and immediately fixed by perfusion in a retrograde manner with 4% paraformaldehyde in phosphate-buffered saline (PBS) followed by immersion fixation in buffered 4% paraformaldehyde. Hearts were kept at 4 °C before the tissue was processed. Lungs were extracted, blotted dry, and weighed. Increased cardiac mass was assessed by comparing the ratios between heart weight-to-tibial length. Pulmonary congestion was assessed by comparing lung weight-to-tibial length ratios.

### Fibrosis Measurement

Extracted hearts were rinsed with PBS, placed in 10% Neutral Buffered Formalin, and paraffin-embedded. Hearts were sliced into 5 µm sections and collagen fibers were stained with Picro-Sirius Red stain. Images were taken with an Olympus BH-2 Bright-field Microscope using the 20x objective lens. Quantification of fibrosis was performed using ImageJ software.

### Cell Isolation

Left ventricular myocytes were isolated using a Langendorff perfusion system^38^. Briefly, mice were anesthetized by inhalation of isofluorane. When the reflexes were absent, hearts were excised, placed into the Langendorff system and perfused with a Ca^2+^-free washing solution, followed by an enzyme solution containing Liberase TM (Research Grade, Roche). Freshly isolated ventricular myocytes were stored at room temperature and used within 8 hours of isolation.

### Mitochondrial Ca^2+^ Uptake

Myocytes plated on laminin-coated coverslips were loaded with X-Rhod-1 AM for 40 minutes at 37 °C^37,68^. Coverslips with the attached cells were placed in a perfusion chamber, and then permeabilized with 50 µg/ml Saponin in the intracellular solution containing (in mM): 135 KCl, 0 NaCl, 20 HEPES, 5 pyruvate, 2 glutamate, 2 malate, 0.5 KH_2_PO4, 0.5 MgCl_2_, 15 2,3-butanedione monoxime, 5 EGTA, and 1.86 CaCl_2_ to yield a free [Ca^2+^]_i_ of 100 nM with pH 7.2. After permeabilization, the bath solution was changed to the same intracellular solution but without saponin, and extramitochondrial Ca^2+^ ([Ca^2 +^]_em_) was elevated to initiate mitochondrial Ca^2+^ uptake. X-Rhod-1 was excited with the 543-nm line of a green HeNe laser, and emitted fluorescence was measured at 552–617 nm. Mitochondrial X-Rhod-1 fluorescence intensity (F) in each experiment was normalized to the level of fluorescence recorded before stimulation (F_0_) but after cell permeabilization. Changes in [Ca^2+^]_m_ are expressed as ΔF/F0, where ΔF = F − F_0_.

### Basal Mitochondrial Ca^2+^ Measurement

Mitochondria were isolated from left ventricles using a standard differential centrifugation method. Briefly, minced hearts were suspended in mitochondrial isolation buffer (IB_1_) in 67 mM sucrose, 50 mM Tris/HCl, 50 mM KCl, 10 mM EDTA, and 0.2% BSA with a pH = 7.2, homogenized with a glass Teflon pestle, and subsequently centrifuged at 700 g for 10 min at 4 °C. The supernatant was collected and then spun at 8000 g for 10 min at 4 °C. The pellet was then resuspended in ice-cold buffer (IB_2_) containing 250 mM sucrose, 3 mM EGTA/Tris, and 10 mM Tris/HCl with a pH = 7.2, followed by centrifugation at 8000 g for 10 min at 4 °C. Mitochondria were then resuspended in IB_2_. Protein concentration was measured using the Bradford Assay method.

Basal mitochondrial calcium was measured in isolated mitochondria using Molecular Devices Spectra Max M5 fluorometer in combination with Ca^2+^ indicator Rhod-2. A calibration curve was generated in a 96-well plate with Ca^2+^ suspended in deionized water, ranging from 0 µM to 3 µM Ca^2+^. 10 µg of isolated mitochondria were spun down at 8000 g at 4 °C for 10 minutes. The IB_2_ was then removed from the pelleted mitochondria, and replaced with water to lyse the mitochondria. All wells were incubated with Rhod-2 for 30 minutes at 37 °C and then measured using a fluorescent plate reader at Ex/Em 552/581 nm.

### ATP Measurements

ATP measurements were performed indirectly via the free magnesium (Mg^2+^) concentration using the fluorescent dye mag-fluo-4 (Invitrogen, ThermoFisher Scientific, Grand Island NY)^37,68^. Since free [Mg^2+^]_i_ is kept constant within a rather narrow range, ATP hydrolysis leads to concomitant increase in free [Mg^2+^]_i_ as measured with fluorescent Mg^2+^ indicators such as mag-fluo-4. Therefore, an increase in mag-fluo-4 fluorescence indicates a decrease in ATP concentration. For ATP measurements myocytes were loaded with 10 µM mag-fluo-4 (λ_ex_ = 488 nm, λ_em_ = 565–605 nm) for 30 min at 37 °C. All data from these measurements are expressed as R = 1 − F/F_0_.

### mPTP activity

mPTP activity was monitored in permeabilized myocytes loaded with 5 μM calcein/AM (λ_ex_ = 488 nm, λ_em_ = 510 nm) for 40 min at 37 °C^37,38^. Opening of mPTP resulted in the loss of mitochondria-trapped calcein (620 Da) and a decrease of fluorescence. At the end of each recording 10 μg/ml of the pore-forming antibiotic alamethicin was applied to provide a control measure for maximum calcein release from mitochondria. Loss of mitochondrial calcein induced by elevating Ca^2+^]_em_ was quantified as the rate of decline of fluorescence (d(ΔF)/dt) calculated from the linear fit to the initial decrease of calcein fluorescence. The rate of decline was normalized (%) to the basal decline of calcein fluorescence addition (taken as 100%) before [Ca^2+^]_em_ elevation.

### ROS Generation

ROS production was measured in intact myocytes loaded with 0.5 μM MitoSox Red (λ_ex_ = 543 nm, λ_em_ = 555–617 nm) for 30 min at 37 °C^37,68^. Changes in MitoSox Red fluorescence intensity (F) were normalized to the level of fluorescence recorded prior to stimulation (F_0_), and expressed as ΔF/F_0_.

### Mitochondrial Redox State

Flavin adenine dinucleotide (FAD)-linked protein autofluorescence (λ_ex_ = 488 nm, λ_em_ = 510 nm) was measured to evaluate mitochondrial redox state^37,38,68^. Data are presented as the ratio of oxidized FAD to reduced FADH_2_ (FAD/FADH_2_) calculated as (F − F_min_)/(F_max_ − F_min_) where F is the fluorescence intensity, and F_min_ is the fluorescence obtained after addition of 4 mM NaCN (inhibits respiration and promotes maximal FAD reduction, i.e. FADH_2_ formation), taken as 0%. F_max_ is the fluorescence obtained after addition of 10 μM FCCP (stimulates maximal respiration, completely oxidizing the mitochondrial FADH_2_ pool), taken as 100%.

### Mitochondrial Membrane Potential

Changes in mitochondrial membrane potential (ΔΨ_m_) were followed using the potential-sensitive dye tetramethylrodamine methyl ester (TMRM; λ_ex_ = 514 nm, λ_em_ = 590 nm)^37,38,68^. Cells were exposed to 5 nM TMRM for 30 min at 37 °C prior to experiments. All solutions contained 5 nM TMRM during recordings.

### Mitochondrial Complex I activity

We used colorimetric assays (Abcam, Cambridge MA) kits for these two complexes. Mitochondria were isolated using the standard procedure stated above. After isolation, proteins were extracted from the mitochondria using the provided detergent solution. Twenty micrograms of proteins were then loaded into wells pre-coated with antibodies specific for Complex I. Assay time, reagents and buffers added, were followed exactly as stated in the protocol booklets for each complex.

### Mitophagy measurements

Adenoviral gene transfer in isolated myocytes that were kept in short-term culture (24–48 h; multiplicity of infection of 500) was used to express mCherry-Parkin and GFP-LC3 to monitor Parkin accumulation in mitochondria and LC3-mediated autophagosome formation^69^. Freshly isolated myocytes were plated on sterile, laminin-coated glass coverslips in PC-1 medium, supplemented with penicillin and streptomycin (50 μg/ml). PINK1, Parkin, SQSTM1/p62, TOMM22 and accumulation of LC3-II were also monitored by Western blot.

### SDS-PAGE and Western Blotting

Primary antibodies for TSPO (Abcam, ab109497), COX IV (Abcam, ab16056), PINK1 (Abcam ab23707), SQSTM1/p62 (Abcam ab56416), TOMM22 (Abcam ab57523), LC3 (MLB, M186-3), Parkin (Santa Cruz Biotechnology sc-133167) and β-Actin (Santa Cruz Biotechnology sc-47778) were diluted to a concentration of 1:1000, and secondary antibodies were conjugated with Horseradish Peroxidase was diluted to a concentration of 1:2000. Signals were detected using an Alpha FluorChem Imaging Systems 8900 and bands were quantified relative to housekeeping protein (Supplementary Fig. 2).

### Immunohistochemistry

Hearts were fixed in 4% PFA and then embedded in paraffin. 5 micrometer sections were cut and these sections were deparaffined in a series of xylene and alcohol concentrations^70^. Antigen retrieval was performed using heat in sodium citrate buffer. Blocking was done at room temperature for 2 hours using TBS with 10% goat serum and 1% BSA. Primary antibodies were incubated at 1:300 overnight. TSPO (red, ABCAM ab109497) and α-actinin (green, Sigma A7811) were the primary antibodies. Images were taken using a Zeiss LSM 700 Confocal Laser Scanning Microscope.

### Statistical analysis

All data are expressed as mean ± standard error. Statistical significance of differences between experimental groups was determined using Student paired *t* test or two-way ANOVA, followed by Tukey post-test when appropriate. A value of p < 0.05 was considered statistically significant. The datasets generated during and/or analyzed during the current study are available from the corresponding author on reasonable request.

## Electronic supplementary material

Supplementary File
