# Supplementary material for: Cardiac-specific Conditional Knockout of the 18-kDa Mitochondrial Translocator Protein Protects from Pressure Overload Induced Heart Failure
Source: Sci Rep. 2018 Nov 1;8:16213. doi: 10.1038/s41598-018-34451-2 (PMC6212397; doi:10.1038/s41598-018-34451-2)

Cardiac-specific Conditional Knockout of the 18-kDa Mitochondrial Translocator Protein Protects from Pressure Overload Induced Heart Failure

Phung N. Thai[1], Daniel J. Daugherty[2], Bert J. Frederich[1], Xiyuan Lu[3], Wenbin Deng[2], Donald M. Bers[3], Elena N. Dedkova[3*], Saul Schaefer[1,4 *]

Supplementary Figure 1A and B.

(A) Immunohistochemistry in the four groups is shown, with staining for TSPO (red), α-actinin (green), DAPI (blue) and merged images. Under WT Sham conditions, TSPO is seen throughout the myocytes with more localization to the peri-myocyte regions (arrows). As expected, the TSPO signal is markedly increased in the WT TAC myocardium, with predominant signal in peri-myocyte regions (dark areas). In both KO sham and KO TAC sections, there is reduced signal intensity of TSPO, consistent with the results of protein electrophoresis (Figure 1G). The localization of TSPO signal again appears to be peri-myocyte, consistent with residual TSPO in non-cardiac cells.

(B) Bottom panels demonstrates that negative controls with secondary antibodies did not show any signal.

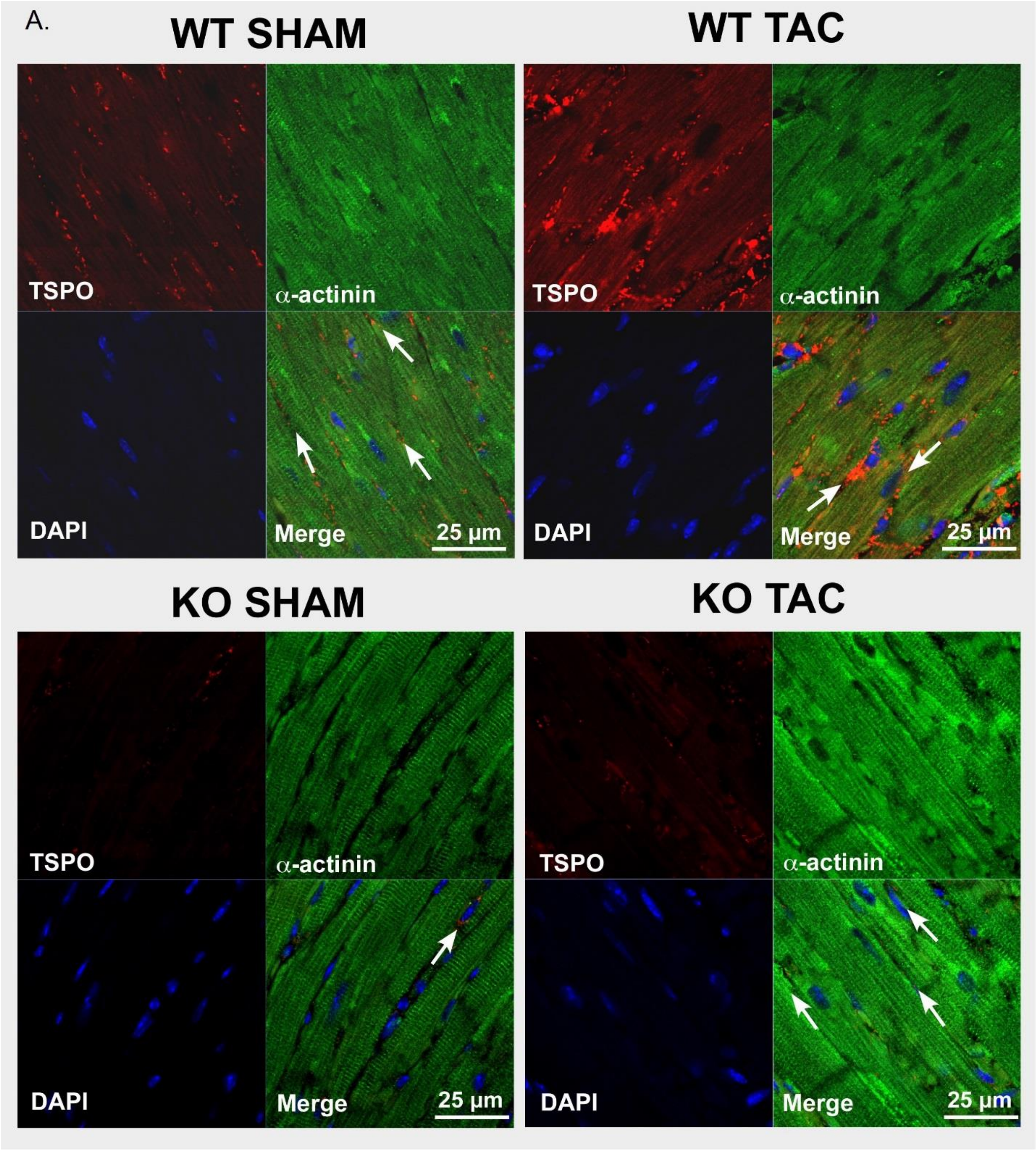
A.
WT SHAM
WT TAC
TSPO
α-actinin
DAPI
Merge
25 µm
KO SHAM
KO TAC

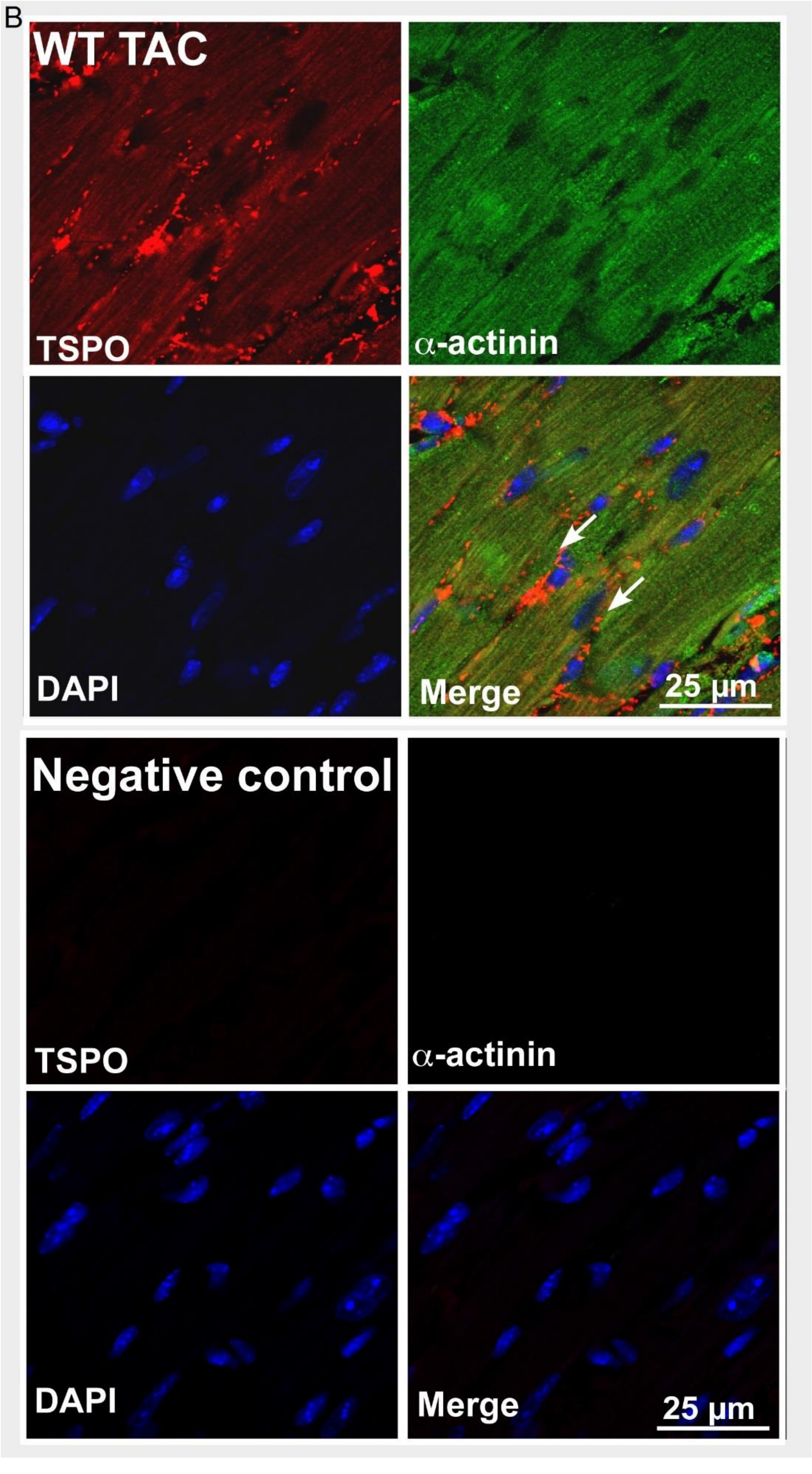
B
WT TAC
TSPO
α-actinin
DAPI
Merge
25 μm
Negative control
TSPO
α-actinin
DAPI
Merge
25 μm

Supplementary Figure 2

Shown below are the original gels with loading controls from the same gel for the illustration in Figure 7D. Samples were derived from whole tissue lysates in the 4 experimental groups. Included are additional bots for TSPO with actin controls, with results similar to those seen in Figure 1F.

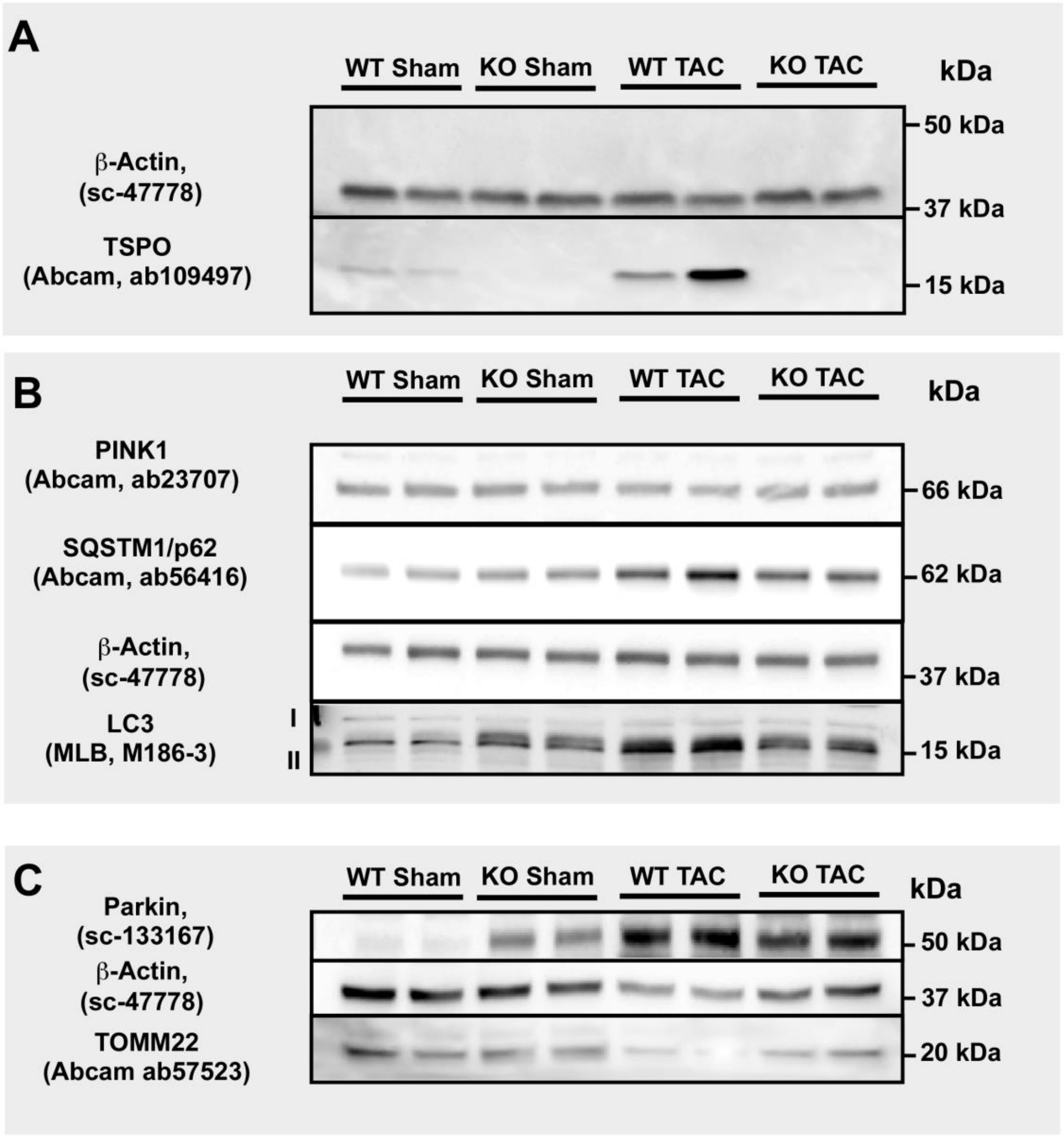

Supplement: Supplementary file 1 — Supplementary File [file 41598_2018_34451_MOESM1_ESM.pdf]
